# Sclerotherapy Versus Transection for Acute Variceal Bleeding

**DOI:** 10.1155/1994/86120

**Published:** 1994

**Authors:** D. Westaby

**Affiliations:** Cross Hospital Fulham Palace Road London W6 8RF UK


*HPB Surgery*, 1994, Vol. 7, pp. 327-340
Reprints available directly from the publisher
Photocopying permitted by license only

© 1994 Harwood Academic Publishers GmbH
Printed in the United States of America

### HPB INTERNATIONAL

**EDITORIAL & ABSTRACTING SERVICE**

JOHN TERBLANCHE, EDITOR
Department of Surgery,
Medical School · Observatory 7925
Cape Town · South Africa
Telephone: (021) 406-6232
Telefax (021) 448-6461

### SCLEROTHERAPY VERSUS TRANSECTION FOR ACUTE VARICEAL BLEEDING

ABSTRACT

*Burroughs, A.K., Hamilton, G. Phillips, A., Mezzanotte, G., McIntyre, N. and Hobbs, K.E.F. (1989) A comparison of sclerotherapy with staple transection of the esophagus for the emergency control of bleeding from esophagus varices. The New England Journal of Medicine; 321: 857–862*

**We compared two procedures for the emergency treatment of bleeding esophageal varices in patients who did not respond to blood transfusion and vasoactive drugs. We randomly assigned 101 patients with cirrhosis of the liver and bleeding esophageal varices to undergo either emergency sclerotherapy (n = 50) or staple transection of the esophagus (n = 51). Four patients assigned to sclerotherapy and 12 assigned to staple transection did not actually undergo those procedures, but all analyses were made on an intention-to-treat basis.**

**Total mortality did not differ significantly between the two groups; the relative risk of death for staple transection as compared with sclerotherapy was 0.88 (95 percent confidence interval, 0.51 to 1.54). Mortality at six weeks was 44 percent among those assinged to sclerotherapy and 35 percent among those assigned to staple transection. Complication rates were similar for the two groups. An interval of five days without bleeding was achieved in 88 percent of those assigned to staple transection and in 62 percent of those assinged to sclerotherapy after a single injection (P 0.01) and 82 percent after three injections. In only 2 of the 11 patients who received a third sclerotherapy injection was bleeding controlled for more than five days, and 9 died.**

**We conclude that staple transection of the esophagus is as safe as sclerotherapy for the emergency treatment of bleeding esophageal varices and that it is more effective than a single sclerotherapy procedure. We currently recommend surgery after two injection treatments have failed. (N Engl. J. Med. 1989; 321: 857–62).**

### PAPER DISCUSSION

KEY WORDS: Sclerotherapy, oesophageal transection, varices, portal hypertension

The paper by Burroughs *et al.*, is one of several important contributions which have originated from this group, concerned with the management of variceal bleeding. The prospective randomised controlled trial in question, aimed to compare injection sclerotherapy with stapling oesophageal transaction for the management of active oesophageal varix bleeding. Clear definitions of eligibility and end-points for the trial were pre-determined, as were the number of patients to be enrolled. The ability to control active bleeding was similar for both treatment groups, as was short and long-term survival. Injection sclerotherapy was associated with a higher frequency of early and late rebleeding.

The failure to show a significant difference in control of bleeding or survival between these two treatment groups is in keeping with other studies that have compared injection sclerotherapy to oesophageal transection or shunt surgery[1,2]. This would appear to suggest a large degree of interchangeability between endoscopic and surgical techniques for managing active bleeding. However, some concern must be expressed as to whether injection sclerotherapy was used optimally. Entry criteria into the trial in question was restricted to patients who continued to bleed over a period up to 5 days after first presentation with evidence of variceal haemorrhage. All patients underwent an initial endoscopic assessment, but this itself did not determine the type or timing of treatment. The patients were then followed and assessed for active bleeding, but were not randomised unless there was evidence of haemodynamically significant bleeding or a minimum of 6 units of blood transfused within a 24-hour period. For bleeding of any less severity the patients were volume repleted and received vaso-active drugs at the discretion of the attending physicians. The fact that many of the patients included in the study had experienced further major bleeding after initial presentation is substantiated by the median blood transfusion before randomisation which was 13 units, ranging between 5 and 34 units of blood. By the time of inclusion in the study, the majority of the patients had received some form of vasoactive drug and/or balloon tamponade. Whether such delayed intervention with injection sclerotherapy might have reduced the efficacy remains speculation, although there is evidence that immediate injection sclerotherapy at the time of the diagnostic endoscopy has a success rate of approximately 85–90%[3,4] higher than observed in the present study. There is also some evidence that prior treatment with vasoactive drugs might also reduce the efficacy of injection sclerotherapy, perhaps by exacerbating mucosal damage[5]. The possibility that delayed intervention might also influence the outcome from oesophageal transection could equally be proposed.

The authors justify delaying intervention on the basis that many patients would be treated unnecessarily as spontaneous cessation of bleeding is not uncommon. However, in the present study, some 60% of patients either continued to bleed or had early rebleeding (justifying randomisation into the study). Early intervention to arrest active bleeding and to prevent early rebleeding would be justified in all cases if such treatment was both safe and effective. Cost implications must also be considered. It may be argued that the diagnostic endoscopy offers the optimum time for early intervention utilising a technique that is relatively safe, effective and cheap. Whether initial treatment should be extended to long-term regimes is a point of contention beyond the scope of the present article.

An extremely important point emphasised by the authors is the definition of treatment failure, particularly with reference to injection sclerotherapy. They have clearly shown that repeated attempts to manage bleeding by sclerotherapy are associated with diminishing returns and it is suggested that 2 attempts at endoscopic treatment is all that is justified before recourse to other measures. The results of stapling transection observed in the present trial would strongly support this technique for the early rescue of patients who have failed endoscopic therapy. Any such intervention should be considered over a period of hours rather than days after the time of presentation. The only reservation with respect to this advice may be the possible adverse effect of staple transection upon any subsequently planned liver transplantation.

**REFERENCES**

1. Huizinga, W., Angorn, P. and Baker, L. (1985) Esophageal transection versus injection sclerotherapy in the management of bleeding esophageal varices in patients at high risk. *Surg. Gynecol Obstet.*, **160**, 539–546
2. Teres, J., Bordas, J.M., Bravo, D. *et al.* (1987) Sclerotherapy vs distal splenorenal shunt in the elective treatment of variceal hemorrhage: A randomized controlled trial. *Hepatology*, **7**, 430–436
3. Paquet, K-J, Feussner, H. (1985) Endoscopic sclerosis and esophageal balloon tamponade in acute hemorrhage from esophagogastric varices: A prospective controlled randomized trial. *Hepatology*, **5**, 580--583
4. Moreto, M., Zaballa, M., Bernal, A. *et al.* (1988) A randomized trial of tamponade or sclerotherapy as immediate treatment for bleeding esophageal varices. *Surg. Gynecol. Obstet.*, **167**, 331--334
5. Westaby, D., Hayes, P., Gimson, A. *et al.* (1989) Controlled clinical trial of injection sclerotherapy for active variceal bleeding. *Hepatology*, **9**, 274–277

Dr D. Westaby
Consultant Physician and Gastroenterologist
Charing Cross Hospital
Fulham Palace Road
London W6 8RF
United Kingdom

### TWO VARIETIES OF INTRAHEPATIC CHOLANGIOCARCINOMA

**ABSTRACT**

*Yamamoto, J., Kosuge, T., Takayama, T., Shimada, K., Makuuchi, M., Yoshida, J., Sakamoto, M., Hirohashi, S., Yamasaki, S. and Hasegawa, H. (1992) Surgical treatment of intrahepatic cholangiocarcinoma: Four patients surviving more than five years. Surgery; 111: 617–622*

